# Comparing skeletal and dentoalveolar effects of fixed palatal crib and bonded spurs in early management of anterior open bite associated with non-nutritive sucking habits: a randomized clinical trial

**DOI:** 10.1007/s40368-025-01086-7

**Published:** 2025-08-01

**Authors:** R. H. Shams, M. M. Ali, N. Kabel, A. H. El Khadem

**Affiliations:** 1 Paediatric Dentistry and Dental Public Health Department, Faculty of Dentistry, Cairo University, Giza, Egypt; 2 Department of Paediatric Dentistry, Misr University for Science and Technology, 6th of October City, Egypt; 3 Paediatric Dentistry and Dental Public Health, Faculty of Dentistry, Cairo University, Giza, Egypt; 4 Paediatric Dentistry and Dental Public Health, Faculty of Dentistry, Misr University of Science and Technology, 6th of October City, Egypt

**Keywords:** Fixed palatal crib, Bonded spurs, Anterior open bite, Non-nutritive sucking habits

## Abstract

**Purpose:**

To evaluate the effects of fixed palatal cribs and bonded spurs on skeletal and dentoalveolar parameters during treatment of anterior open bite associated with non-nutritive sucking habits.

**Methods:**

This randomized clinical trial involved 30 children aged 6–11 years with an anterior open bite ≥ 1 mm. Participants were assigned randomly to two groups: fixed palatal crib (FPC, *n* = 15, mean age 8.6 years) and bonded spurs (BS, *n* = 15, mean age 8.3 years). Digital bio-models and lateral cephalometric images were obtained at baseline and after 12 months of treatment. Paired and independent *t* tests were used for intra- group and inter-group comparisons, respectively.

**Results:**

The FPC group had significantly greater overbite correction than the BS group (1.22 ± 2.18 mm, *p* = 0.00387), with 73.33% achieving a positive overbite compared to 6.67% in the BS group. The FPC group also showed significantly higher increases in mandibular arch perimeter (2.57 ± 0.79 mm, *p* = 0.00314), maxillary arch length (1.56 ± 0.35 mm, *p* = 0.00013), mandibular arch length (1.62 ± 0.30 mm, *p* = 0.00001), and intermolar width (0.71 ± 0.24 mm, *p* = 0.00001). Cephalometric findings showed greater reduction in the lower central incisor mandibular plane angle (6.75° ± 1.65°, *p* = 0.00032) and a corresponding increase in the interincisal angle (6.12° ± 2.61°, *p* = 0. 02654), while skeletal measurements showed no statistically relevant variation between the two groups.

**Conclusion:**

The fixed palatal crib appliance was more effective than bonded spurs in managing anterior open bite in growing patients, mainly via dentoalveolar changes, (such as incisor retroclination), without affecting skeletal parameters.

## Introduction

Anterior open bite (AOB) refers to a type of malocclusion characterized by a lack of vertical overlap between the upper and lower incisors despite the posterior teeth maintaining occlusal contact (Tavares and Allgayer 2019). It can result from a combination of skeletal factors (e.g., excessive vertical facial growth) and environmental influences such as prolonged non-nutritive sucking habits or tongue thrusting (Ngan and Fields 1997). In Egypt, AOB prevalence ranges between 1.6 and 20.9%, depending on age and gender (ElMotaleb et al. 2014; Fsifis et al. 2016; El-Sayed and Abdel Ghani 2019). Severity is classified by the vertical separation between incisors: moderate (0–2 mm), severe (3–4 mm), and extreme when exceeding 4 mm (Proffit et al. 2007). Anterior open bite is associated with a substantially increased risk of speech articulation errors (occurring 12–50 times more often than normal) and a marked reduction in mastication efficiency (approximately 60–72%) (Tavares and Allgayer 2019). Relapse after correction is also common; approximate 10–30% of cases tend to redevelop the AOB over time (González Espinosa et al. 2020).

Treatment approaches for AOB are generally categorized into three strategies: eliminating the etiologic habit (if present), correcting dental malocclusion, or addressing skeletal discrepancies through orthopedic or surgical means, depending on the underlying cause and severity (Wang et al. 2024). In habit-related AOB diagnosed early, initial therapy often consists of non-appliance methods, such as behavior modification and positive reinforcement (Davidson 2008; Borrie et al. 2015). If these methods fail to eliminate the habit, habit-breaking appliances (e.g., palatal cribs or lingual spurs) are recommended to facilitate bite closure and correct tongue positioning (Huang et al. 1990; Justus 2001).

The fixed palatal crib (FPC) was first introduced in the 1950s (Massler and Chopra 1950). It facilitates spontaneous bite closure and allows normal incisor eruption (Berger and Janisse 2013; Leite et al. 2016). However, palatal cribs have been associated with speech difficulties, hygiene issues, and tongue discomfort (Iqbal et al. 2019). Bonded spurs (BS), introduced in 2005, are a less intrusive alternative; they are attached to the lingual or palatal surfaces of the incisors (Nogueira et al. 2005). Bonded spurs guide tongue posture through proprioceptive feedback, modifying tongue function without significantly obstructing the oral space (Justus 2001).

Both appliances serve as mechanical reminders to interfere with improper tongue posture (Huang et al. 1990; Justus 2001). However, long-term stability may be jeopardized if underlying orofacial dysfunctions (such as low tongue resting posture or tongue-thrust swallowing) persist after treatment (Smithpeter and Covell 2010). Specifically, the smooth contour of palatal cribs may permit passive tongue contact, limiting neuromuscular re-education (Artese et al. 2011). Consequently, orofacial myofunctional therapy (OMT) has been recommended as an adjunctive strategy to reinforce neuromuscular adaptation and establish proper tongue posture and swallowing patterns, thereby enhancing the stability of results (Smithpeter and Covell 2010; Chandel et al. 2024).

A systematic review by Koletsi et al. (2018) emphasized the absence of robust evidence regarding early orthodontic interventions for AOB, and concluding that bonded spurs have no proven superiority over fixed banded appliances. Similarly, Meng et al. (2022) found only two studies directly comparing FPC and BS, with limited and inconclusive evidence on their long-term outcomes and adverse effects. Given these gaps, this trial aims to examine comprehensively the effects of FPC and BS on both skeletal and dentoalveolar structures, incorporating detailed analyses of arch dimensions and vertical skeletal changes in children with AOB associated with non-nutritive sucking habits (NNSHs).

## Materials and methods

The randomized clinical trial (parallel groups) was conducted in the Outpatient Diagnostic Clinic, Department of Paediatric Dentistry and Dental Public Health, Faculty of Dentistry, Cairo University, from September 2022 to May 2023. Ethical approval was obtained from the Research Ethics Committee of the Faculty of Dentistry, Cairo University (approval date 28/06/2022). The trial protocol was prospectively registered at ClinicalTrials.gov (Identifier: NCT05313399).

### Sample size calculation

Sample size determination was conducted through a power analysis targeting differences in AOB correction between the FPC and BS groups. Based on a previous trial by (Leite et al. 2016), the analysis incorporated an alpha level of 0.05, 80% statistical power, and an effect size of 1.23 (Cohen’s *d*), derived from changes in overbite correction. The smallest required sample size was calculated to be 24 participants. An additional 25% was added to account for potential dropouts, resulting in a final sample of 30 children, with 15 assigned randomly to each group. Sample size estimation was performed using G*Power (version 3.1.9.7) (Faul et al. 2007).

### Inclusion criteria

Children aged 6–11 years with an anterior open bite ≥ 1 mm associated with NNSHs, and adaptive tongue thrust were included. Participants were required to have fully erupted permanent upper and lower central incisors and a clinically confirmed Angle Class I molar relationship.

### Exclusion criteria

Children were excluded if they had craniofacial anomalies (e.g., cleft lip and/or palate, craniosynostosis, or syndromic conditions affecting craniofacial growth), any developmental or systemic disorders, a history of orthodontic treatment, or prior extraction of permanent teeth due to trauma or caries lesions.

### Subject recruitment

**Fig. 1:**
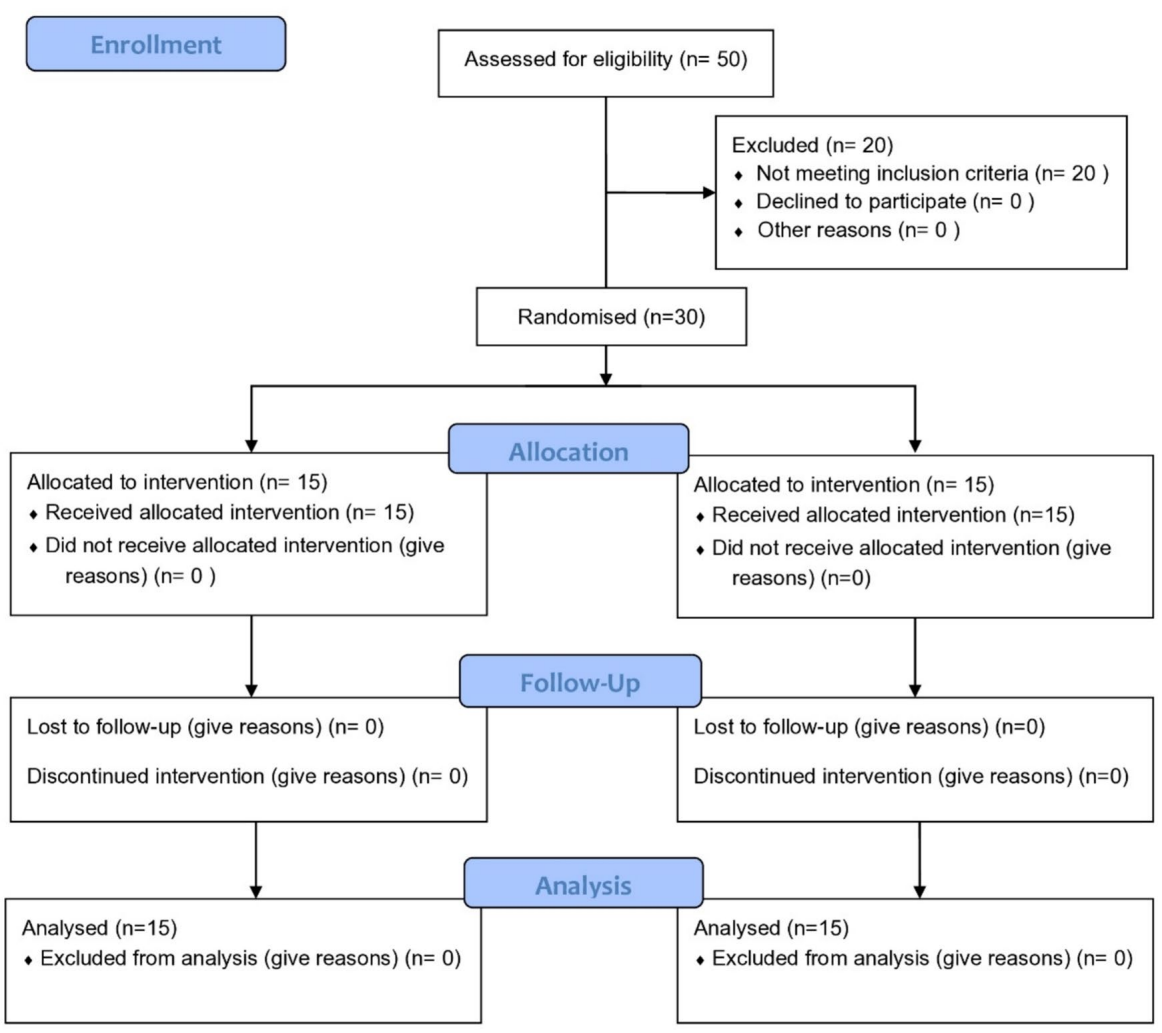
CONSORT flow chart

Recruitment was conducted between September 2022 and May 2023. A total of 50 participants were initially screened for eligibility, 20 did not meet the predefined inclusion criteria and were therefore excluded. The remaining 30 eligible participants were allocated randomly into two groups: the FPC group and the BS group, with 15 children in each. The randomization sequence was generated by M.M.A using www.random.org, and sealed opaque envelopes were prepared for allocation concealment, and R.H conducted group assignments, recorded participant details, and managed follow-up coordination. The randomization protocol and study design were developed and approved by A.H, who ensured proper methodological planning and compliance. Follow-up was completed by May 2024 following the scheduled course of treatment. Participant flow is illustrated in the CONSORT flow chart (Fig. 1). All 30 participants completed the 1-year follow-up, with no dropouts.

### Clinical examination

Before treatment, participants underwent comprehensive documentation, including intraoral and extraoral photographs, panoramic radiographs, digital lateral cephalometric radiographs, and digital bio-models were generated by scanning stone dental casts using the Medit i500 scanner (Medit Corp., Seoul, South Korea) to produce stereolithographic (STL) files for model analysis. The clinical examination evaluated AOB, molar relationship, tongue thrust, and oral habits. A preparatory behavioral phase provided each child with oral hygiene instruction and caries control measures.

### Blinding

Outcome assessors were blinded to group assignments; however, blinding was not feasible for the operator or patients due to the visible nature of the appliances.

### Interventions

Bonded spurs (Tongue Tamers®, Ortho Technology, Tampa FL) were placed on the palatal surfaces of the maxillary incisors and the lingual surfaces of the mandibular incisors, at the cervical and incisal thirds of the teeth, respectively, to reduce potential occlusal interference. Attachment of the spurs was achieved using Transbond™ XT (3M Unitek, St. Paul, MN, USA), a light-cured orthodontic adhesive after acid etching to ensure optimal retention (Justus 2001; Leite et al. 2016; Aliaga-Del Castillo et al. 2021a, b).

The FPC was fabricated by adapting orthodontic bands (Ormco Corporation, Glendora, CA, USA) with a 0.9 mm wire loop soldered to extend between the maxillary canines. Orthodontic separators were used prior to cementation to ensure a proper fit. The appliance was cemented with glass ionomer cement (Medicem glass ionomer, Promedica Dental Material GmbH, Germany) was used for secure attachment (Leite et al. 2016). Participants who did not show adequate AOB improvement or required additional treatment were referred for comprehensive orthodontic therapy.

### Outcome measures

**Fig. 2:**
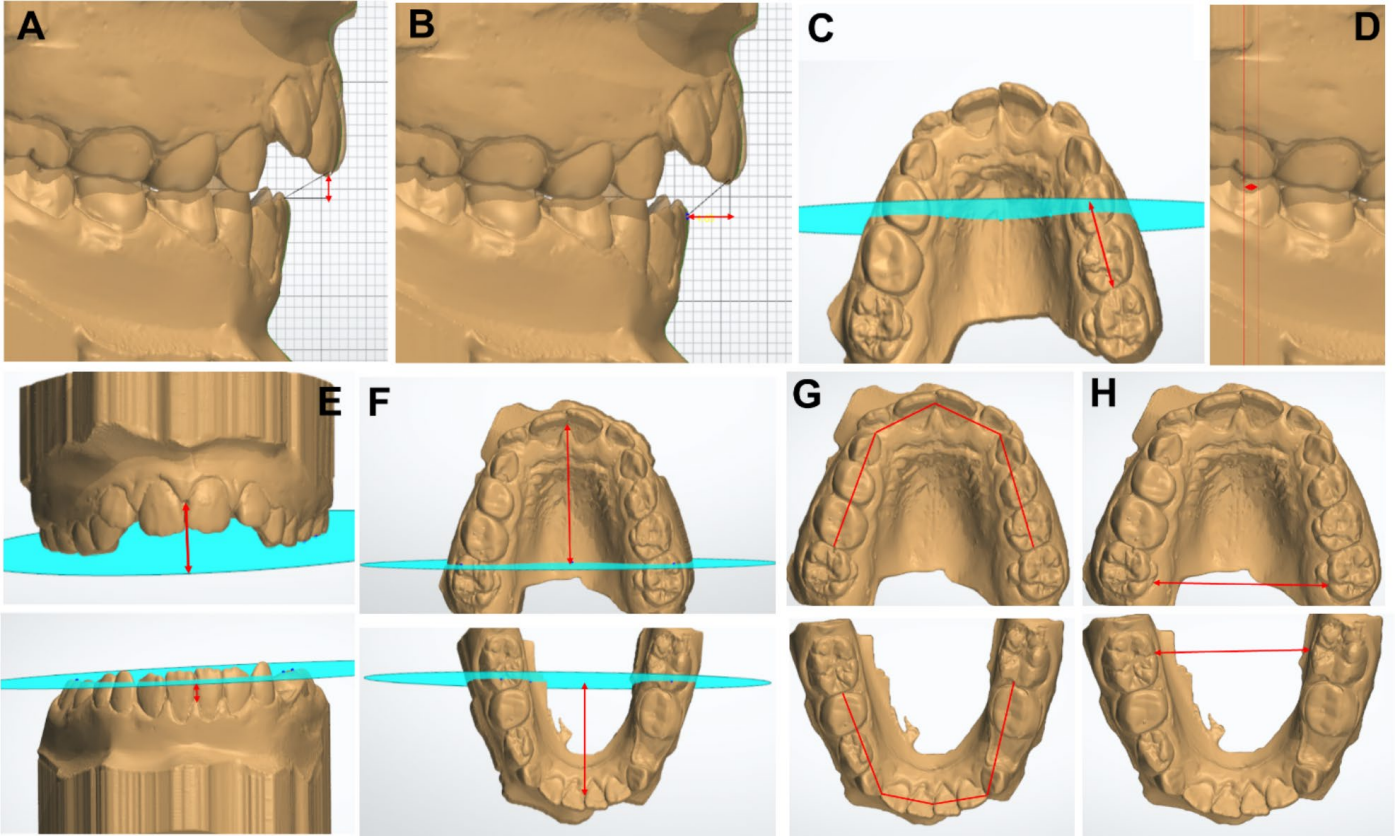
Digital model illustrations showing the measured dental variables **A** Overbite. **B** Overjet. **C** Antero-posterior position of the upper first molar. **D** Molar relationship. **E** Vertical dental development. **F** Maxillary and mandibular arch length. **G **Maxillary and mandibular arch perimeter. **H** Intermolar width

**Fig. 3:**
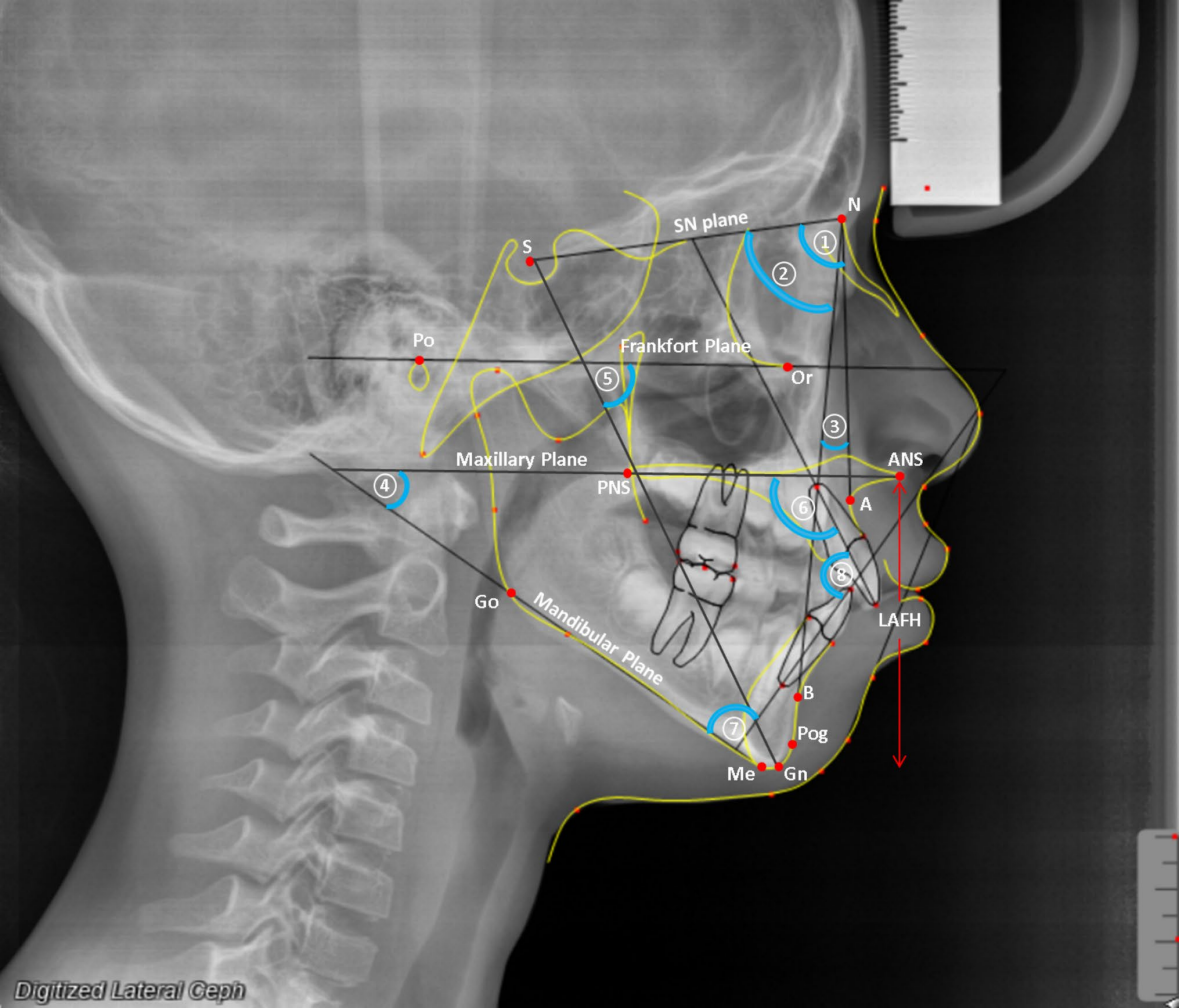
Lateral cephalometric tracing illustrating anatomical landmarks and skeletal and dentoalveolar angular measurements used in orthodontic analysis. The key cephalometric points include: *S* Sella, *N* Nasion, *A* A point, *B* B point, *Pog* Pogonion, *Gn* Gnathion, *Me* Menton, *Go* Gonion, *ANS* anterior nasal spine, *PNS* posterior nasal spine, *Or* orbitale, *Po* porion and *LAFH* lower anterior facial height and numbered angular variables include 1. *SNA* Sella–Nasion–A point angle, 2. *SNB* Sella–Nasion–B point angle, 3. *ANB* A point–Nasion–B point angle, 4. *MMPA* maxillary–mandibular plane angle, 5. *Y*-axis, 6. *Mx1.PP* maxillary central incisor to palatal plane angle, 7. *IMPA* mandibular central incisor to mandibular plane angle and 8. 1.1 interincisal angle

The primary outcome was overbite correction, measured in millimeters from digital bio-models. A secondary outcome included achievement of a positive overbite (incisal overlap) as a clinical success indicator (Slaviero et al. 2017; Aliaga-Del Castillo et al. 2021a, b) Other outcomes included changes in arch dimensions and vertical skeletal relationships. Digital bio-models and vertical skeletal changes were assessed through lateral cephalograms. Digital bio-models were analyzed with OrthoAnalyzer 3D software (3Shape, Copenhagen, Denmark) for overbite, arch length, arch perimeter, vertical dental development, and intermolar distance (Fig. 2). Lateral cephalometric analysis was performed with Dolphin Imaging Software Version 11.5 (Dolphin® Imaging and Management Solutions, Patterson Dental Supply, Inc., Chatsworth, CA) to assess skeletal and dentoalveolar changes. The maxillo-mandibular plane angle (MMPA) and other angular and linear variables were measured (Fig. 3). The detailed description of all measured variables is provided in (Table 1). All measurements were made by R.H. and independently verified by A.H., both of whom were trained and calibrated in the software.

**Table 1:** Linear measurements on digital bio-models

Measurement type	Variables	Definition
Digital cast measurements	Dental relationship, mm	
	Overbite	The vertical overlap is measured from the incisal edge of the upper central incisor to the corresponding surface of the lower incisor
	Overjet	The horizontal gap between the labial surface of the upper incisor and that of the lower incisor, assessed in a parallel plane to the occlusal surface
	(U6-rugae)	Distance from the mesial surface of the upper first molar (U6) to a line intersecting the right and left third palatal rugae
	Molar relationship	Mean horizontal distance between the mesiobuccal cusp of upper first molar and the mesiobuccal groove of lower first molar (right and left sides)
	Vertical development, mm	
	Mx and Md anterior dentoalveolar vertical development	The vertical measurement represents the perpendicular span from the alveolar ridge at the contact point (or midpoint in cases of spacing) between the central incisors, extending occlusally in a frontal projection. The occlusal reference plane was determined by connecting the mesiobuccal cusps of the permanent first molars with the cusp tips of either the deciduous first molars or the first premolars on each side of the maxilla and mandible
	Arch dimensions, mm	
	Arch perimeter	The total arch length is determined by summing four linear segments starting at the mesial surface of the permanent first molars, extending through the deciduous canines to the central incisors on each side
	Arch length	The shortest linear distance drawn perpendicularly from a horizontal line connecting the mesial aspects of the first molars to the point of contact, or midpoint, between the central incisors at the gingival margin
	6-6	The linear distance between the first permanent molars measured at the midpoint of the palatal or lingual gingival margin
Lateral cephalometric measurements	Dental relationship	
	1.1	This angle is measured at the point where the long axes of the upper and lower central incisors intersect
	Maxillomandibular relationship	
	ANB (°)	Calculated by subtracting the SNB angle from the SNA angle, this measurement indicates the antero-posterior positional relationship between the maxilla and mandible
	Vertical components	
	*Y*-axis (°)	The line connecting Sella to Gnathion is an estimate of the direction of mandibular growth
	LAFH (mm)	Represents the vertical facial height measured between the anterior nasal spine (ANS) and menton (Me), indicating the vertical dimension of the lower facial third
	MMPA	The angular relationship between the palatal plane (spanning from ANS to PNS) and the mandibular plane (from Gonion to Menton), reflecting the vertical skeletal pattern
	Maxillary and mandibular dentoalveolar components	
	Mx1.PP (°)	This is the angular relationship between the upper incisor’s axis and the palatal plane, which reflects the degree of maxillary incisor inclination
	IMPA (°)	The angular distance between the axis of the lower central incisor and the mandibular plane, indicating the extent of incisor proclination or retroclination

U6-rugae: antero-posterior position of the upper first molar, Mx Ver: maxillary vertical development, Md Ver Mandibular vertical development, 6-6: inter first permanent molars width, 1.1: interincisal angle, ANB: A point–Nasion–B point angle, LAFH: lower anterior facial height, MMPA: maxillary–mandibular plane angle, Mx1.PP: maxillary central incisor to palatal plane angle and IMPA: mandibular central incisor to mandibular plane angle

### Data processing and analysis

Statistical analyses were performed using SPSS® (version 20, IBM Corp., Armonk, NY, USA). Data normality was evaluated using both the Kolmogorov–Smirnov and Shapiro–Wilk tests, confirming that all variables followed a normal distribution in both groups, as confirmed by non-significant *p* values (*p* > 0.05) supporting the use of parametric analysis. Equality of variances was assessed using Levene’s test, which indicated homogeneity across all comparisons. Given that the assumptions of normality and homogeneity of variances were met, parametric tests were appropriately employed. Paired *t* tests were used for within-group comparisons and independent *t* tests for between-group comparisons (significance set at *p* < 0.05). Categorical outcomes (e.g., treatment success defined as positive overbite) were compared using the chi-square test. Relative risk (RR) with 95% confidence intervals was calculated. Intra- and inter-observer reliability were assessed by re-evaluating 10% of the measurements 1 month after initial analysis; intraclass correlation coefficients (ICCs) were 0.93 (intra-observer) and 0.91 (inter-observer), indicating excellent agreement. No changes were made to the trial outcomes after commencement.

## Results

### Baseline data

Demographic characteristics, such as age and gender, for both groups are presented in (Table 2).

**Table 2:** The baseline demographic data, including age and gender, for each group

Demographic data	FPC	BS	*p *value
Gender no. (%)			
Male	5 (33.3%)	5 (33.3%)	1.000
Female	10 (66.7%)	10 (66.7%)	1.000
Mean age (years ± SD)	8.60 ( ± 1.55)	8.33 (± 0.98)	0.591

### Numbers analyzed

**Fig. 4:**
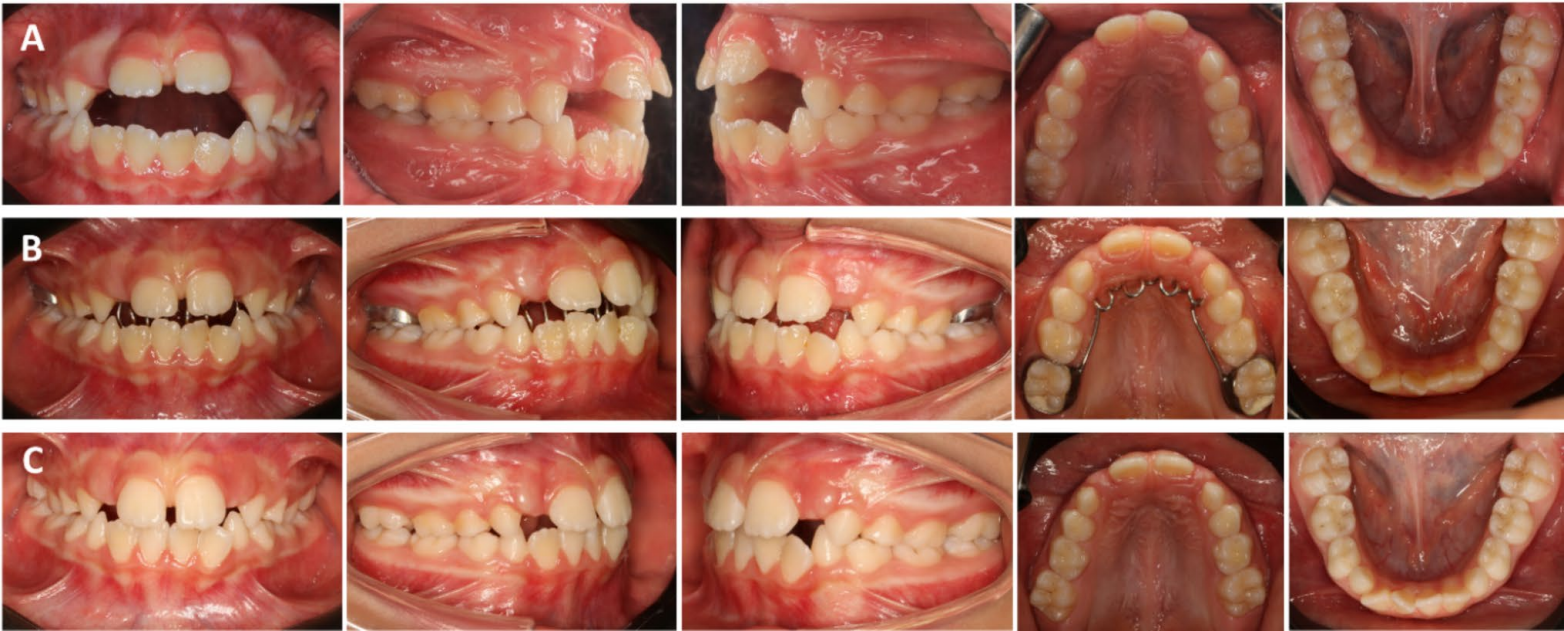
**A** Intraoral pre-treatment pictures of FPC group patient. **B** Pictures taken in the middle of treatment. **C** Post-treatment

**Fig. 5:**
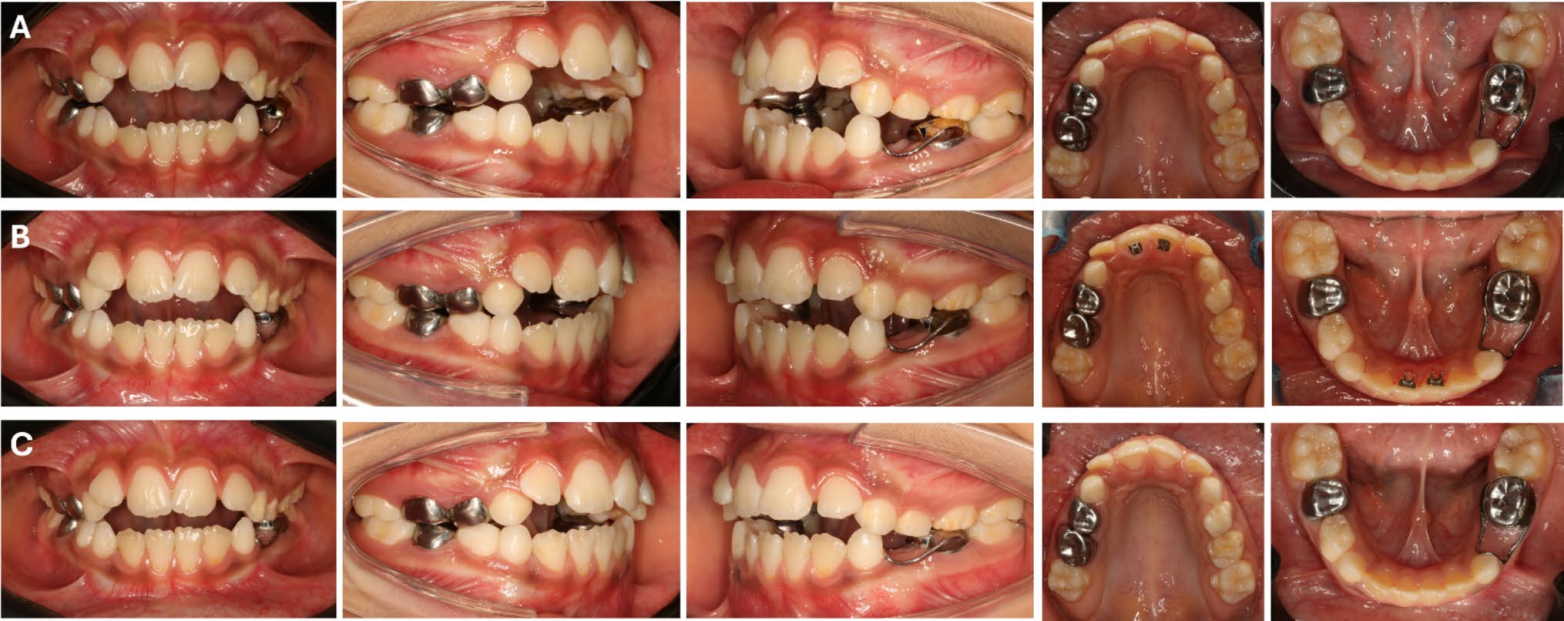
**A** Intraoral pre-treatment pictures of BS group patient. **B** Pictures taken in the middle of treatment. **C** Post-treatment

At 12 months post-treatment, digital bio-model evaluation revealed notable alterations within the FPC group. Overbite increased significantly by 3.11 ± 0.93 mm (Fig. 4), with a mesial movement of the upper first molars (1.22 ± 0.83 mm), while the sagittal molar relationship remained unchanged (0.29 mm, *p* > 0.05). Additionally, arch dimensions were reduced, with mandibular arch perimeter (MdAP), maxillary arch length (MxAL), mandibular arch length (MdAL), and maxillary intermolar width distance (Mx6-6) decreasing by 2.46 ± 1.51 mm, 1.43 ± 1.22 mm, 1.27 ± 1.27 mm, and 0.55 ± 0.62 mm, respectively (Table 3). These dentoalveolar modifications, identified through digital bio-models as changes in tooth position and alveolar bone without skeletal base involvement, contributed to a significantly higher proportion of patients achieving a positive overbite in the FPC group (73.33%) compared to the BS group (6.67%), indicating that patients treated with FPC were 11 times more likely to achieve overbite correction, with a relative risk of 11.0 (95% CI 1.62–74.88). In contrast, the BS group exhibited a significant increase in overbite by 1.89 ± 1.00 mm (Fig. 5), while maxillary vertical development showed a significant decrease by 0.98 ± 1.04 mm (Table 4).

**Table 3:** Pre- and post-treatment means, standard deviations (SD), and the outcomes of paired *t *tests assessing the treatment-related differences in the FPC group

Measurement type	Variables	Pre-treatment		Post-treatment		Change		95% CI for the change		*p* value
		Mean	SD	Mean	SD	Mean	SD	Lower bound	Upper bound	
Digital cast measurements	Dental relationship									
	Overbite (mm)	− 2.41	1.99	0.71	1.88	3.11	0.93	2.78	3.44	0.00155**
	Overjet (mm)	2.67	1.69	2.82	1.91	0.15	1.36	− 0.60	0.91	0.34021
	U6-rugae (mm)	11.3	2.33	10.11	2.43	− 1.22	0.83	− 1.68	− 0.76	0.01998*
	Molar relationship	1.13	0.85	1.43	1.11	0.29	1.45	0.51	1.10	0.44659
	Vertical development									
	Mx Ver (mm)	7.61	1.92	6.57	1.32	− 1.04	2.10	− 2.20	0.12	0.07553
	Md Ver (mm)	3.66	1.98	3.37	1.71	− 0.28	0.93	− 0.80	0.23	0.25359
	Arch dimension									
	MxAp	74.88	3.09	75.49	6.83	0.61	7.11	− 3.33	4.55	0.74382
	MdAP	69.16	3.29	66.70	3.59	− 2.46	1.51	− 3.29	− 1.62	0.00002***
	MxAL	27.23	1.32	25.80	1.86	− 1.43	1.22	− 2.10	− 0.75	0.00046***
	MdAL	23.27	1.57	22.00	1.75	− 1.27	− 1.27	− 1.64	− 0.90	0.00000***
	Mx6-6	34.60	2.70	34.04	2.66	− 0.55	0.62	− 0.90	− 0.21	0.00392**
	Md6-6	32.62	1.80	32.66	1.74	0.04	0.47	− 0.22	0.30	0.76674
Lateral cephalometric measurements	Dental relationship									
	1.1 (°)	109.38	10.10	115.77	10.37	6.39	6.77	2.64	10.14	0.00260**
	Maxillomandibular relationship									
	ANB (°)	5.99	2.43	6.13	2.21	0.13	1.10	− 0.48	0.74	0.64610
	Vertical components									
	*Y*-axis (°)	70.39	3.14	69.51	3.76	− 0.88	2.33	− 2.17	0.41	0.64610
	MMPA (°)	30.57	3.69	30.99	3.13	0.42	2.68	− 1.06	1.90	0.55299
	LAFH (mm)	60.41	4.37	61.47	3.94	1.07	4.10	− 1.20	3.34	0.33068
	Dentoalveolar measurements									
	Mx1.PP (°)	119.73	5.49	120.10	5.66	0.37	5.00	− 2.39	3.14	0.77647
	IMPA (°)	100.27	6.22	93.14	6.22	− 7.13	4.86	− 9.83	− 4.44	0.00006***

*CI* confidence interval

*p* values interpretation: *p ≤ 0.05: significant; **p ≤ 0.01: highly significant; ***p ≤ 0.001: very highly significant; ns: non-significant (*p* > 0.05)

**Table 4:** Pre- and post-treatment means, standard deviations (SD), and the outcomes of paired *t *tests assessing the treatment-related differences in the BS group

Measurement type	Variables	Pre-treatment		Post-treatment		Change		95% CI for the change		*p* value
		Mean	SD	Mean	SD	Mean	SD	Lower bound	Upper bound	
Digital cast measurements	Dental relationship									
	Overbite (mm)	− 3.57	1.68	− 1.68	1.89	1.89	1.00	1.53	2.25	0.00000***
	Overjet (mm)	2.40	1.89	2.55	1.45	0.15	0.88	− 0.34	0.64	0.81086
	U6-rugae (mm)	11.47	2.05	11.27	1.95	− 0.21	1.06	− 0.80	0.38	0.78825
	Molar relationship	1.17	0.57	1.24	0.51	0.07	0.71	− 0.32	0.46	0.70431
	Vertical development									
	Mx Ver (mm)	7.80	1.50	6.82	2.16	− 0.98	1.04	0.27	− 1.56	0.00254**
	Md Ver (mm)	4.52	1.28	4.24	1.26	− 0.27	1.34	− 1.02	0.47	0.44328
	Arch dimension									
	MxAp	76.55	3.49	76.40	3.62	− 0.15	1.27	− 0.86	0.55	0.44328
	MdAP	69.16	3.89	69.27	3.47	0.11	2.68	− 1.38	1.59	0.87747
	MxAL	27.64	1.79	27.77	1.60	0.13	0.60	− 0.20	0.46	0.42043
	MdAL	23.13	1.79	23.48	1.82	0.35	0.96	− 0.18	0.88	0.17724
	Mx6-6	34.64	2.58	34.79	2.52	0.16	0.70	− 0.23	0.55	0.39676
	Md6-6	32.33	2.24	32.33	2.41	0.00	0.66	− 0.36	0.36	0.99691
Lateral cephalometric measurements	Dental relationship									
	1.1 (°)	112.21	9.35	112.48	8.89	0.27	7.52	− 3.89	4.44	0.89008
	Maxillomandibular relationship									
	ANB (°)	6.03	1.77	5.75	2.60	− 0.28	1.29	− 0.99	0.43	0.41474
	Vertical components									
	*Y*-axis (°)	71.04	2.88	71.35	2.20	0.31	1.55	− 0.54	1.17	0.44580
	MMPA (°)	33.51	3.59	32.37	4.50	− 1.14	3.89	− 3.29	1.01	0.27522
	LAFH (mm)	61.82	4.47	61.56	3.78	− 0.26	3.52	− 2.21	1.69	0.77917
	Dentoalveolar measurements									
	Mx1.PP (°)	117.28	6.15	118.64	5.69	1.36	8.06	− 3.11	5.83	0.52424
	IMPA (°)	97.00	5.42	96.62	6.15	− 0.38	4.13	− 2.67	1.91	0.52424

* CI* confidence interval

*p* values interpretation: *p ≤ 0.05: significant; **p ≤ 0.01: highly significant; ***p ≤ 0.001: very highly significant; ns: non-significant (*p* > 0.05)

Lateral cephalometric analysis confirmed significant dentoalveolar changes in both groups. In the FPC group, a significant reduction in incisor mandibular plane angle (IMPA) by 7.13° ± 4.86° was observed, accompanied by an increase in the interincisal angle (1.1) by 6.39° ± 6.77°, reflecting significant incisor inclination changes (Table 3). In the BS group, no cephalometric variables showed statistically significant changes (Table 4).

Comparative analysis (Table 5) revealed that the FPC group achieved greater improvements in overbite correction compared to the BS group, with a mean difference of 1.22 ± 2.18 mm. Additionally, IMPA reduction was significantly greater in the FPC group by 6.75° ± 1.65°, indicating a more pronounced effect on incisor inclination and overall dentoalveolar changes. The FPC group also demonstrated a significantly greater increase in the interincisal angle (6.12° ± 2.61°) compared to the BS group. Regarding arch dimensional changes, the FPC group exhibited significantly greater reductions, with MdAP decreasing by 2.57 ± 0.79 mm, MxAL by 1.56 ± 0.35 mm, and MdAL by 1.62 ± 0.30 mm. Additionally, the Mx6-6 was reduced by 0.71 ± 0.24 mm. However, skeletal parameters, including MMPA, exhibited no statistically significant differences found between groups, with a minimal mean change of 1.56° ± 1.22°, indicating no significant alterations in vertical skeletal relationships across treatment modalities.

**Table 5:** Mean values, standard deviations (SD), and outcomes of independent *t* tests comparing changes across the two groups

Measurement type	Variables	FPC-BS		95% CI for the change		*p* value
		Mean	SD	Lower bound	Upper bound	
Digital cast measurements	Dental relationship					
	Overbite (mm)	1.22	2.18	0.44	2.00	0.00387**
	Overjet (mm)	0.01	0.42	− 0.85	0.87	0.92783
	U6-rugae (mm)	− 1.01	1.23	− 1.69	− 0.34	0.01097*
	Molar relationship	0.22	1.62	− 0.67	1.12	0.59928
	Vertical development					
	Mx Ver (mm)	− 0.06	0.60	− 1.29	1.18	0.99691
	Md Ver (mm)	− 0.01	0.42	− 0.87	0.85	0.99691
	Arch dimension					
	MxAp	0.76	1.87	− 3.06	4.58	0.68547
	MdAP	− 2.57	0.79	− 4.19	− 0.94	0.00314**
	MxAL	− 1.56	0.35	− 2.27	− 0.84	0.00013***
	MdAL	− 1.62	0.30	− 2.24	− 1.00	0.00001***
	Mx6-6	− 0.71	0.24	− 1.21	− 0.22	0.00652**
	Md6-6	0.04	0.21	− 0.39	0.46	0.85891
	Dental relationship					
	1.1 (°)	6.12	2.61	0.77	11.47	0.02654*
Lateral cephalometric measurements	Maxillomandibular relationship					
	ANB (°)	0.41	0.44	− 0.48	1.31	0.35323
	Vertical components					
	*Y*-axis (°)	− 1.19	0.72	− 2.67	0.29	0.10996
	MMPA (°)	1.56	1.22	− 0.94	4.06	0.21103
	LAFH (mm)	1.33	1.40	− 1.53	4.19	0.21103
	Dentoalveolar measurements					
	Mx1.PP (°)	− 0.99	2.45	− 6.00	4.03	0.69013
	IMPA (°)	− 6.75	1.65	− 10.13	− 3.38	0.00032***

*CI* confidence interval

*p* values interpretation: *p ≤ 0.05: significant; **p ≤ 0.01: highly significant; ***p ≤ 0.001: very highly significant; ns: non-significant (*p* > 0.05)

### Harms

In the FPC group, some patients reported discomfort due to embedding of the appliance into the soft palate, leading to soft tissue pain and injury. In the BS group, debonding occurred in 86.67% of patients, with an average of 0.87 debonding events per patient throughout the trial period. Some spurs were swallowed during meals, raising concerns among patients. Additionally, plaque accumulation around the spurs was observed despite oral hygiene instructions.

## Discussion

Prolonged NNSHs can lead to dentomaxillofacial abnormalities such as an AOB (Artese et al. 2011). Early intervention is crucial to intercept and correct these habits, preventing further dental arch and occlusal deviations (Otaren et al. 2021). Habit-breaking appliances, such as lingual spurs and palatal cribs, act as effective intraoral reminders to discourage the habit (Khayami et al. 2013; Reddy and Dawjee 2019). A systematic review (Meng et al. 2022) identified only four studies evaluating the effects of FPC and BS in managing AOB, with just two directly comparing these appliances. Most existing research has focused primarily on overbite correction, while changes in arch dimensions and vertical skeletal changes remain insufficiently investigated. Given these gaps, this trial provides a comprehensive evaluation of the skeletal and dentoalveolar impacts of FPC and BS, offering clinicians evidence-based guidance for selecting the most effective approach in early AOB intervention.

The statistical analysis showed that, although both FPC and BS groups had significant overbite improvements, the FPC group was significantly more effective, allowing the rejection of the null hypothesis. This trial considered the achievement of a positive overbite as a clinically acceptable indicator of successful AOB correction. Although the FPC group showed superior success compared to the BS group, the overall percentages were lower than those reported by Leite et al. (2016), who documented a 100% success rate with FPC and 53.8% with BS. Rossato et al. (2018) also reported modest success across appliance types, suggesting that differences in appliance design, and follow-up periods may account for these variations.

The effectiveness of FPC can be attributed to its design, which blocks tongue projection and disrupts harmful oral habits (Leite et al. 2016). In contrast, the lesser success in the BS group may be due to the absence of a sharpening procedure for the spurs, as recommended by Justus (2001). This procedure was not implemented due to the absence of standardized guidelines and concerns over potential tongue injury. As a result, we postulate that patients gradually adapted to the spurs, reducing their effectiveness over time as the tongue gets accustomed to the spurs. Thus, the secondary tongue thrusting may not have been resolved over time with BS. Furthermore, the high debonding rate of BS (86.67%) in present sample introduced practical challenges, as frequent appliance detachment likely compromised compliance and treatment stability. Finally, some participants in the BS group may have continued sucking habits despite pre-treatment counseling, further undermining the success of the BS intervention.

Previous studies have reported overbite improvements of 3.95 mm for FPC and 3.07 mm for BS (Leite et al. 2016), as well as 3.60 mm for FPC and 3.09 mm for BS (Rossato et al. 2018), indicating generally comparable performance between both appliances in AOB correction. Although BS produced a statistically significant overbite improvement in this trial, the degree of correction was notably lower than the values of 4.26 mm and 4.38 mm reported by Canuto et al. (2016) and Aliaga-Del Castillo et al. (2021a, b), respectively. This difference may be attributed to the intermittent mode of action of BS, which relies primarily on proprioceptive feedback rather than continuous mechanical interference, as with FPC.

Previous studies (Canuto et al. 2016; Leite et al. 2016; Rossato et al. 2018) reported no significant changes in skeletal variables, aligning with our findings of minimal skeletal effects and suggesting that observed changes are more dentoalveolar than skeletal. However, Mousa et al. (2021) observed a significant reduction in MMPA by 6.69° involving the use of a tongue crib in combination with a removable posterior bite plane, emphasizing how treatment approaches and appliance types may impact skeletal modifications.

The FPC group showed a significantly greater reduction in IMPA, reinforcing previous findings of 5.51° (Leite et al. 2016) and 5.52° (Rossato et al. 2018) but demonstrating an even more substantial effect in this trial. This notable retroclination of the mandibular incisors can be attributed to unopposed pressure from the lower lip during swallowing, a mechanism reinforced by the crib appliance’s design, which restricts tongue thrusting and promotes incisor repositioning. The BS group, however, showed minimal IMPA changes, even lower than past studies; 1.93° (Leite et al. 2016) and 0.91° (Rossato et al. 2018), reinforcing concerns about its limited ability to control incisor inclination. This reduction in the FPC group contributed to an increase in the interincisal angle, aligning with prior reports of 9.65° (Rossato et al. 2018), reflecting its role in improving dental alignment. In contrast, the BS group did not show meaningful interincisal angle changes, contrary to earlier reports of 3.34° (Leite et al. 2016), suggesting greater variability in its clinical performance than previously assumed.

Mesial displacement of the upper first molars was also noted in the FPC group, likely resulting from sustained tongue pressure exerted behind the appliance loops. This pressure-induced shift aligns with previous findings by Fouda et al. (2022) and reinforces the influence of tongue posture on dental arch adaptations. Additionally, reductions in MdAP, MxAL, and MdAL align with previous findings linking these changes to molar repositioning and lower incisor retroclination (Fouda et al. 2022). In contrast, the BS group showed only minor, non-significant alterations, indicating a limited effect on arch dimensions. Notably, while Fouda et al. (2022) reported slight increases in maxillary intermolar distance, the trial findings suggest a reduction in this parameter within the FPC group, possibly due to mesial molar movement.

Despite significant anterior movement of the upper molars in the FPC group (U6-rugae measurements), the sagittal molar relationship remained stable, suggesting a concurrent mesial shift of the lower molars preserving the inter-arch molar relationship. This mandibular molar movement may be facilitated by normal loss of Leeway space (greater in the mandible) during early mixed dentition (Moyers 1988).

### Limitations

Some limitations should be acknowledged. First, the absence of a non-treated control group limits the ability to distinguish treatment effects from normal craniofacial growth; however, ethical considerations prevent withholding treatment for AOB in growing patients. Second, the lack of a standardized sharpening protocol for BS may have affected their functional efficiency, as all appliances were delivered unmodified due to safety concerns and the absence of established clinical guidelines.

Practical challenges during use were also noted: BS showed frequent detachment, which may have reduced their clinical effectiveness, whereas the FPC required more maintenance, potentially affecting patient comfort. These factors could influence the level of patient cooperation and overall treatment outcomes.

Another limitation is the absence of a formal assessment of tongue posture or classification of AOB phenotypes. Given the role of tongue posture in AOB etiology and relapses, such assessments could have enhanced the interpretation of treatment responses. Finally, while the 12-month follow-up offered meaningful short-term findings, it was insufficient to evaluate long-term treatment stability and relapse risk.

## Conclusions

The FPC appliance demonstrated superior outcomes in overbite correction and dentoalveolar changes compared to BS, while neither appliance significantly affected skeletal parameters. Future research should develop safe, standardized protocols (e.g., spur sharpening) to optimize BS efficacy, and include longer-term follow-up to assess post-treatment stability and soft tissue adaptation.

## Data Availability

Data generated or analyzed in this study can be accessed upon reasonable requests from the corresponding author.

## Declarations

### Trial registration

This trial was registered at clinical trial.gov, ID: NCT05313399.
